# A rare and extensive summer bloom enhanced by ocean eddies in the oligotrophic western North Pacific Subtropical Gyre

**DOI:** 10.1038/s41598-017-06584-3

**Published:** 2017-07-24

**Authors:** Chun Hoe Chow, Wee Cheah, Jen-Hua Tai

**Affiliations:** 1 Department of Oceanography, National Sun Yat-sen University, Kaohsiung, Taiwan; 2 Research Center for Environmental Changes, Academia Sinica, Taipei, Taiwan

## Abstract

The North Pacific Subtropical Gyre (NPSG) is the largest ecosystem on Earth, and it plays a critical role in global ocean productivity and carbon cycling. Here, we report a rare and striking ~2000-km-long phytoplankton bloom that lasted over one month in the western part of the NPSG in summer 2003. The bloom resulted from the co-occurrence of a northward-shifted North Equatorial Current (NEC) supplying additional phosphate, and strong eddy activity that fueled productivity and spread chlorophyll mainly through horizontal stirring. The extensive one-month bloom had a maximum Chl concentration of six times the summer mean value and collectively fixed an additional five teragrams (5 × 10^12^ g) of carbon above the summer average. An increase in the *p*CO_2_ during the bloom suggests that most of the additionally fixed carbon was rapidly consumed.

## Introduction

The western part of the North Pacific Subtropical Gyre (wNPSG, 130°E to 180°E centred along 22°N) is a hotspot of eddies^1^. The eddies are often observed as a long west-east band generated by shearing activity between the eastward-flowing Subtropical Countercurrent (STCC) and the westward-flowing North Equatorial Current (NEC)^2–4^. As a result of the beta planetary effect, the eddies move westward at a speed of approximately 0.1 m/s^2, 5, 6^. Seasonally, the eddies are more active in spring and summer than in other seasons^7^. The eddies were more active from 1996 to 1998 and from 2003 to 2008 on an interannual time scale^3^. In particular, the eddies in 2003 were the strongest among the years from 1998 to 2009 due to a strong STCC enhanced by a surface Ekman temperature gradient convergence within the STCC region^3^.

In the wNPSG, a vertical entrainment associated with a mixed-layer deepening balances the annually integrated surface thermal forcing^8^. The mixed layer depth is generally deeper than 100 m in winter and shallower than 30 m in summer^9^. Strong vertical stratification in summer inhibits the mixing of the surface water with the deep, nutrient-rich water. These conditions result in a chronically nutrient-limited surface layer, making the NPSG the least biologically productive region per unit area in the global ocean^10^.

**Figure 1:**
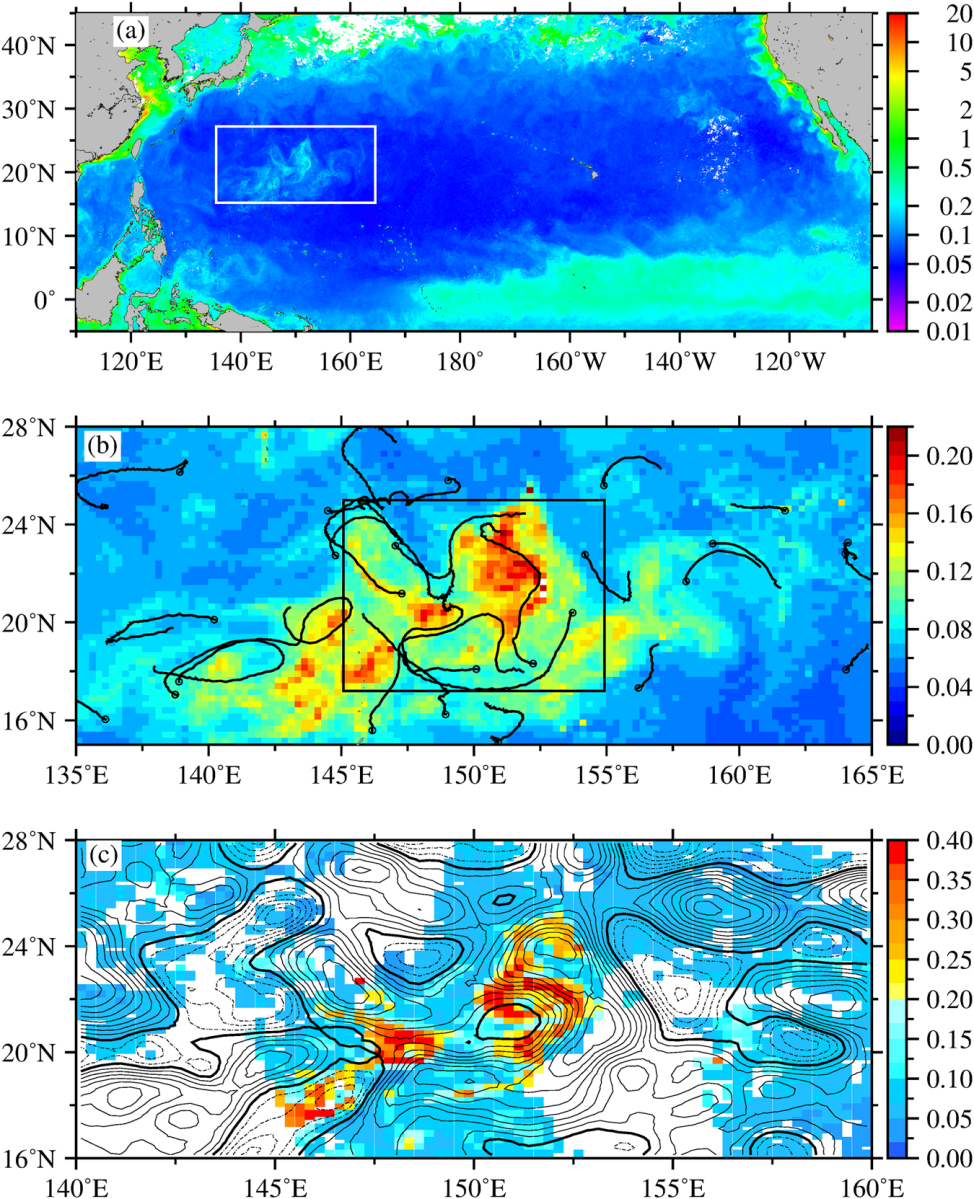
(**a**) Satellite Chl concentration (mg/m^3^) for the NPSG in July 2003 with white bracket showing the bloom area located at the western NPSG. (**b**) Closed up map of the bloom area from July 4 to August 4, 2003. The black curves in (**b**) show the trajectories of surface Argos drifters from July 4 to August 4 of 2003, and the circles at one end of the curves show the drifter locations on July 4. (**c**) SSHAs (contour interval: 4 cm, dashed: negative, solid: positive, bold solid: zero) and Chl (colors) on July 12, 2003. This figure was generated using open source Generic Mapping Tools^33^ (Ver. 5.3.0 http://gmt.soest.hawaii.edu/).

Despite being nutrient-limited in summer, phytoplankton blooms have been reported in the central and eastern part of the NPSG^11–13^. The mechanisms driving these blooms include nitrogen fixation by diazotrophs^14, 15^, eddy interactions^16^, internal waves^17^, and mixing at the subtropical front^18^. To date, there are no reports of large-scale summertime phytoplankton blooms in the wNPSG in the literature. Chlorophyll (Chl) concentrations observed from satellite ocean colour images in the wNPSG are usually lower than 0.06 mg/m^3^ in summer^19, 20^. In July 2003, a huge bloom over ~2000 km long with Chl concentrations >0.2 mg/m^3^ occurred within the area bounded by 135°E to 165°E and 15°N to 28°N, as revealed by satellite images (Fig. 1). The one-month bloom began around July 4 and reached its peak on July 12, 2003, with a Chl concentration of 0.4 mg/m^3^, which is at least six times higher than the summer mean value from 1998 to 2014 (Fig. 1b,c).

We utilized a combination of satellite and *in situ* observations to investigate the mechanisms driving this extraordinary bloom and its fate. The datasets include satellite-derived Chl and aerosol optical thickness (AOT), altimetry-derived eddy kinetic energy (EKE), Argo floats, surface drifters, conductivity-temperature-depth (CTD), surface seawater nutrient and partial pressure of CO_2_ (*p*CO_2_) data. As the Chl blooms resemble rings of eddy-like patterns and the EKE was particularly strong in 2003, we first examined the co-variability of the Chl and eddies and the intensity of the eddies in 2003 compared to other years. From the Argo and CTD’s vertical temperature profiles, we demonstrate that there was a possible deepening of the vertical mixing when the bloom occurred in the wNPSG in 2003. We also analysed the drifter data and performed an eddy composite analysis to show that the stirring induced by the strong eddy currents was the main mechanism sustaining the summertime bloom.

## Results

### Co-variability of the Chl and Eddies

**Figure 2:**
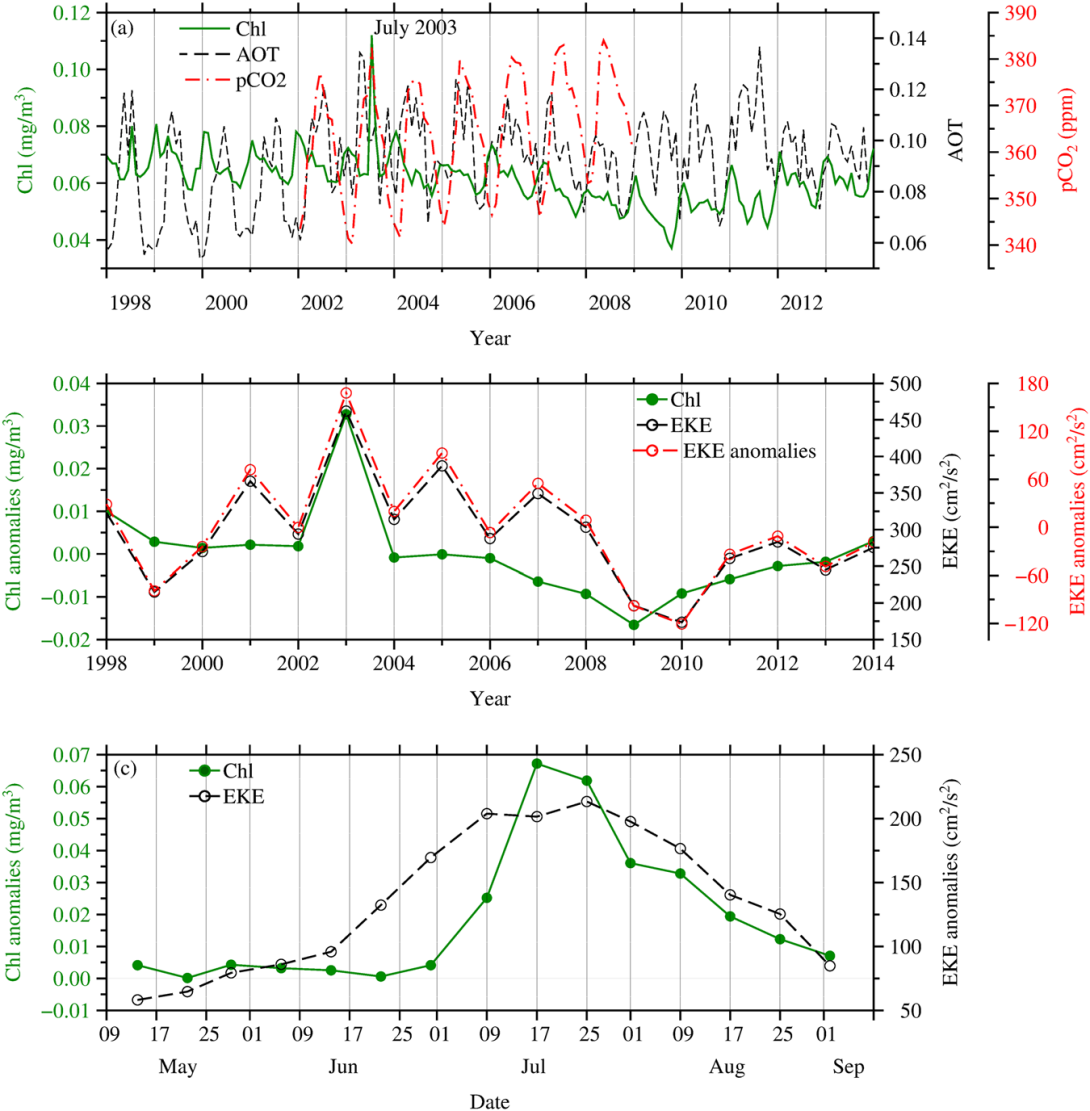
Time series of (**a**) monthly mean Chl, AOT and *p*CO_2_, (**b**) Chl concentration anomalies, EKE means and EKE anomalies in July and August from 1998 to 2014 and (**c**) anomalies of EKE and Chl concentration from May 10 to Sep. 13, 2003, within 145–155°E and 17–25°N.

**Figure 3:**
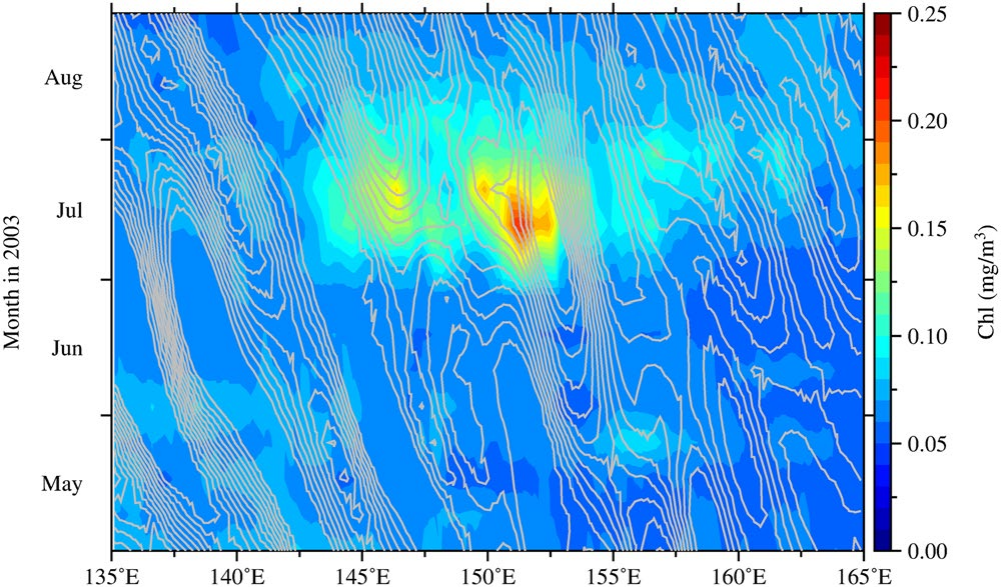
*Hovmöller* diagram of Chl (color) and positive SSH anomalies (contour interval: 2 cm), averaged within 15–28°N.

Considering only the average values within the strongest bloom region (145–155°E and 17–25°N, black box in Fig. 1b), both the EKE and Chl were highest in summer 2003 within the 17-year study period (Fig. 2a,b). The Chl concentrations reached 0.112 mg/m^3^ in July 2003 (Fig. 2a) with an anomaly of 0.05 mg/m^3^. As expected, a significant positive correlation (*r* = 0.7) was observed between the time series of the EKE and Chl (Fig. 3b). Although the EKE was also high in 2001 and 2005, there was virtually no corresponding response in the Chl. The uniqueness of the 2003 bloom can be attributed to (1) an extremely strong EKE in 2003 that was about 20% higher than that in 2001 and 2005, in which the EKE anomaly in 2003 was twice as strong as in 2001 and 2005, and (2) the encapsulation of high-phosphate NEC waters, which extended farther north in 2003 than in other years, by strong eddy stirring (see further explanation in the following section). Further analysis of the EKE and Chl from May 10 to September 13, 2003, showed a time lag (correlation *r* = 0.8) between the EKE and Chl of approximately 2 weeks (Fig. 2c), which is the time required by the eddies to spread the high Chl waters to the east and west within 10 degrees with an eddy current speed of approximately 0.35 m/s obtained from drifter trajectories. The strong correlation between the EKE and Chl suggests that the strong eddies in summer 2003 played a critical role in driving the Chl bloom in summer 2003.

### Roles of Eddy Stirring

In Fig. 1c, at approximately 20°N and 148°E on July 12, the peak of the Chl concentrations resides at the eddy edges between two northwest and southeast anticyclonic eddies. To the east of 150°E, the waters with high Chl concentrations typically circle around the eddy edges between the cyclonic and anticyclonic eddies, located approximately from 20°N to 22°N and 23°N to 25°N, respectively. This eddy-like pattern of the Chl clearly shows the effect of eddy stirring.

In Fig. 1b, the drifter trajectories from July 4 to August 4, 2003, show the corresponding eddy-like patterns of the Chl. Estimated from these drifter data, the surface currents have a mean speed of approximately 0.35 m/s and a standard deviation of approximately 0.20 m/s. The current speed can even reach as high as 1 m/s. Based on the *Hovmöller* diagram shown in Fig. 3, the westward-propagating motion of the sea-surface-height anomalies (SSHAs) can be clearly observed, whereas the Chl distribution does not show a corresponding motion. This finding is in contrast to the findings of Chelton *et al*.^21^, who showed that the phytoplankton bloom moves with the westward motion of the SSHAs. In the present study, the bloom was spreading in two directions: eastward and westward from the core of the bloom at approximately 151°E (Fig. 3). With a speed of 0.35 m/s, the high Chl waters can be transported to a distance of approximately 900 km to the east and west (approximately 1800 km in total) within one month, roughly reaching the zonal range of the bloom within 143°E and 160°E (Fig. 3). Thus, the stirring induced by the anomalous fast eddy currents in the west-east band of the eddy system is strongly suggested to play a key role in spreading the Chl far to the east and west up to a few thousand kilometres.

**Figure 4:**
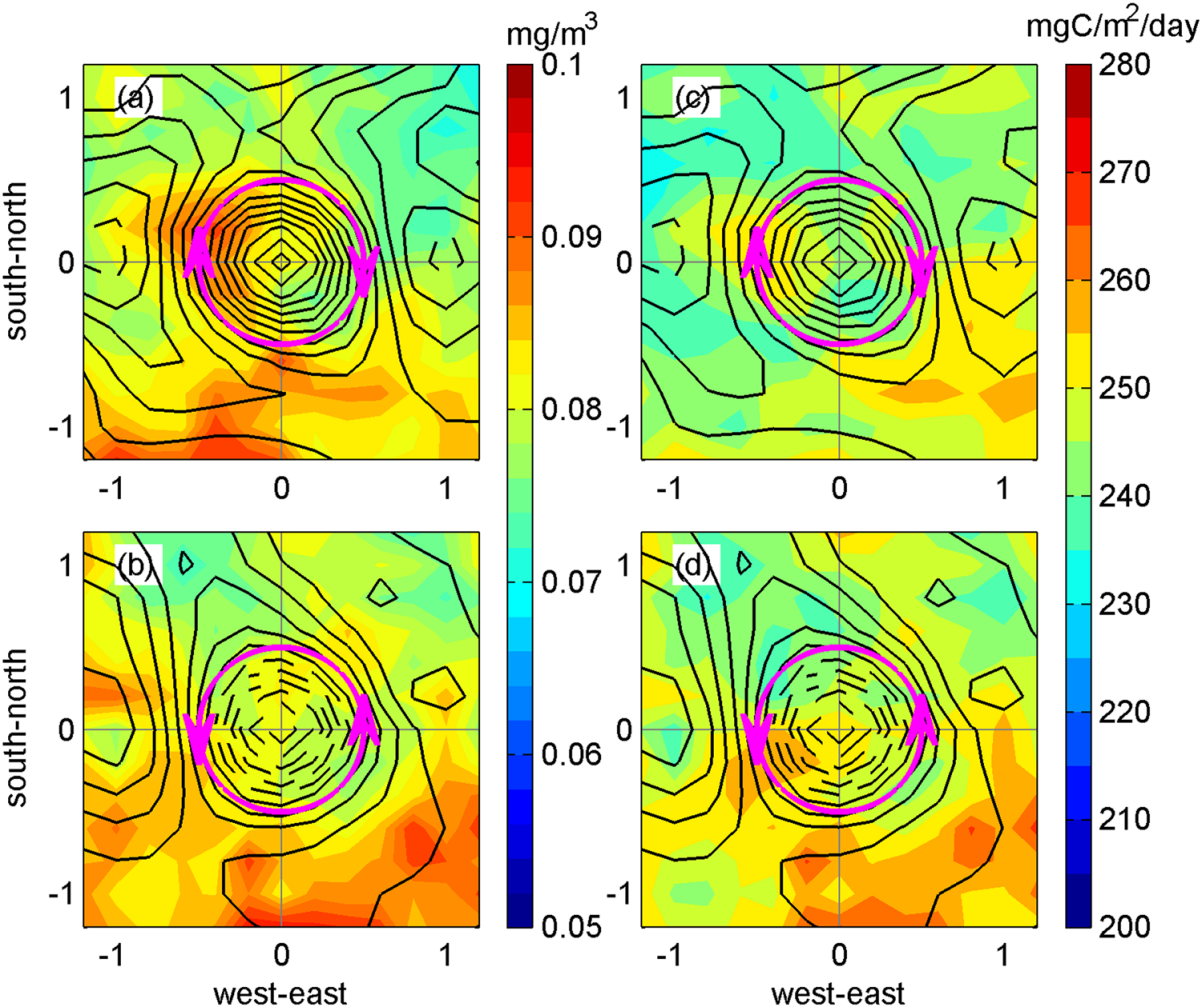
Composite of Chl (left panels) and NPP (right panels) for anticyclonic (**a** and **c**) and cyclonic (**b** and **d**) eddies within 135–165°E and 15–28°N in July 2003. The x- and y-axes are the normalized distance from eddy cores with grid resolution of 0.2. All values are significantly different from means (0.06 mg/m^3^ for Chl and 203 mg C/m^2^/day for NPP) at the 95% confidence level based on *t*-testing.

**Figure 5:**
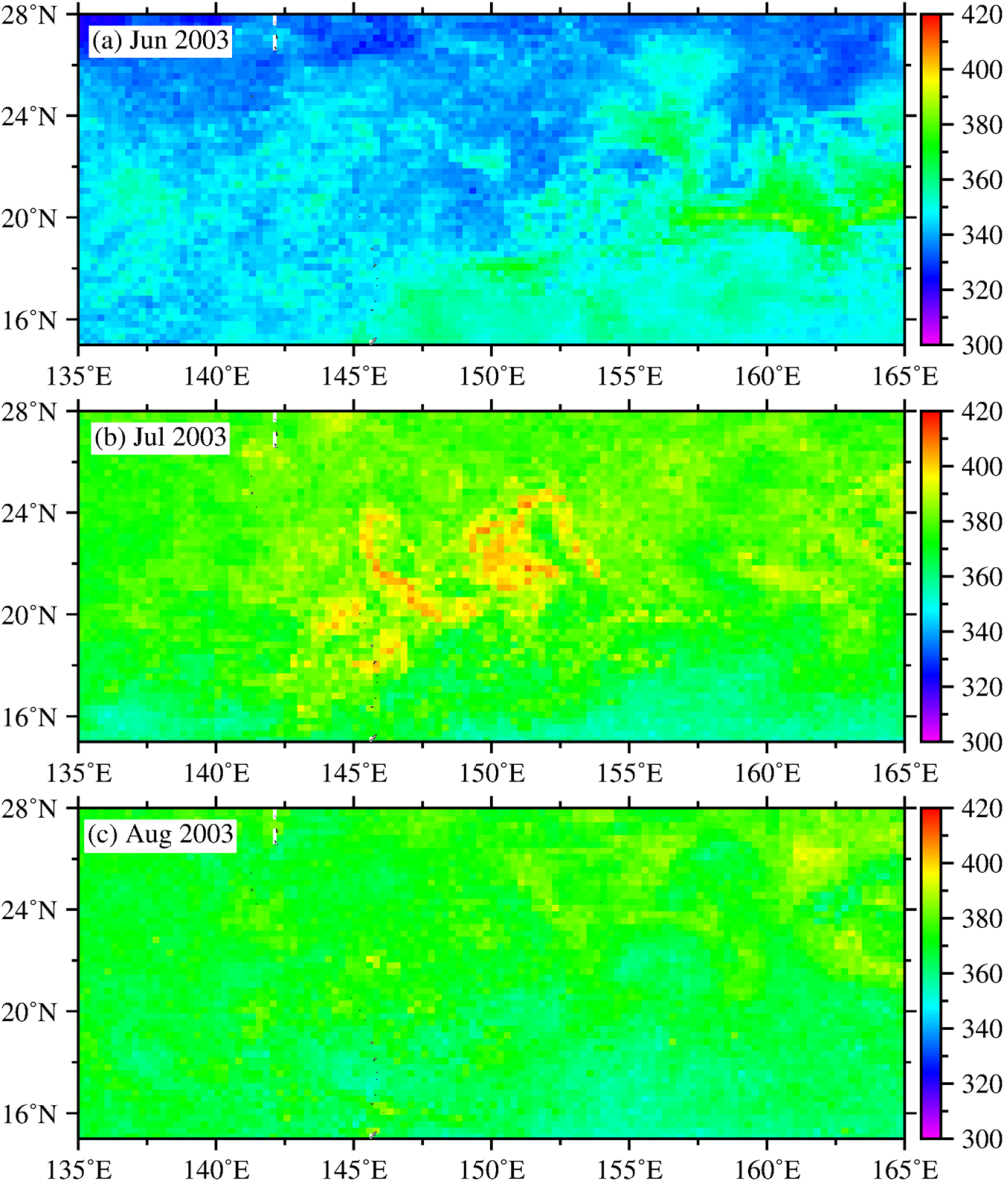
Surface water *p*CO_2_ concentrations (ppm) before (June), during (July), and after (August) the bloom.

Figure 4 shows the composites of 78 anticyclonic eddies (upper panel) and 78 cyclonic eddies (lower panel) for the Chl and net primary production (NPP) within 135–165°E and 15–28°N in July 2003. The SSHA contours in Fig. 4a show the pattern of the anticyclonic eddies at the centre, accompanied by two cyclonic eddies to the west and east, which is the opposite of the eddy pattern shown in Fig. 4b. The Chl concentrations reached 0.1 mg/m^3^ in both the cyclonic and anticyclonic composites and were generally higher at the southern part of the eddies. In Fig. 4a, the horizontal advection of the Chl can be observed at the western edge of the anticyclonic eddies, circling around eddy cores and extending from the south. In Fig. 4b, the high Chl concentrations can also be found around the eddy cores. Corresponding to that of the Chl, a high NPP also resides near the eddy edges, reaching 250 mg C/m^2^/day (Fig. 4c & d). Integrated within 135–165°N and 15–28°N, the extensive one-month bloom collectively fixed an additional five teragrams of carbon, which is approximately 14% of the summer average. However, the concomitant increase in the *p*CO_2_ during the bloom period shown in Fig. 3a and Fig. 5 indicates that this additional input of carbon was rapidly consumed and respired as CO_2_.

**Figure 6:**
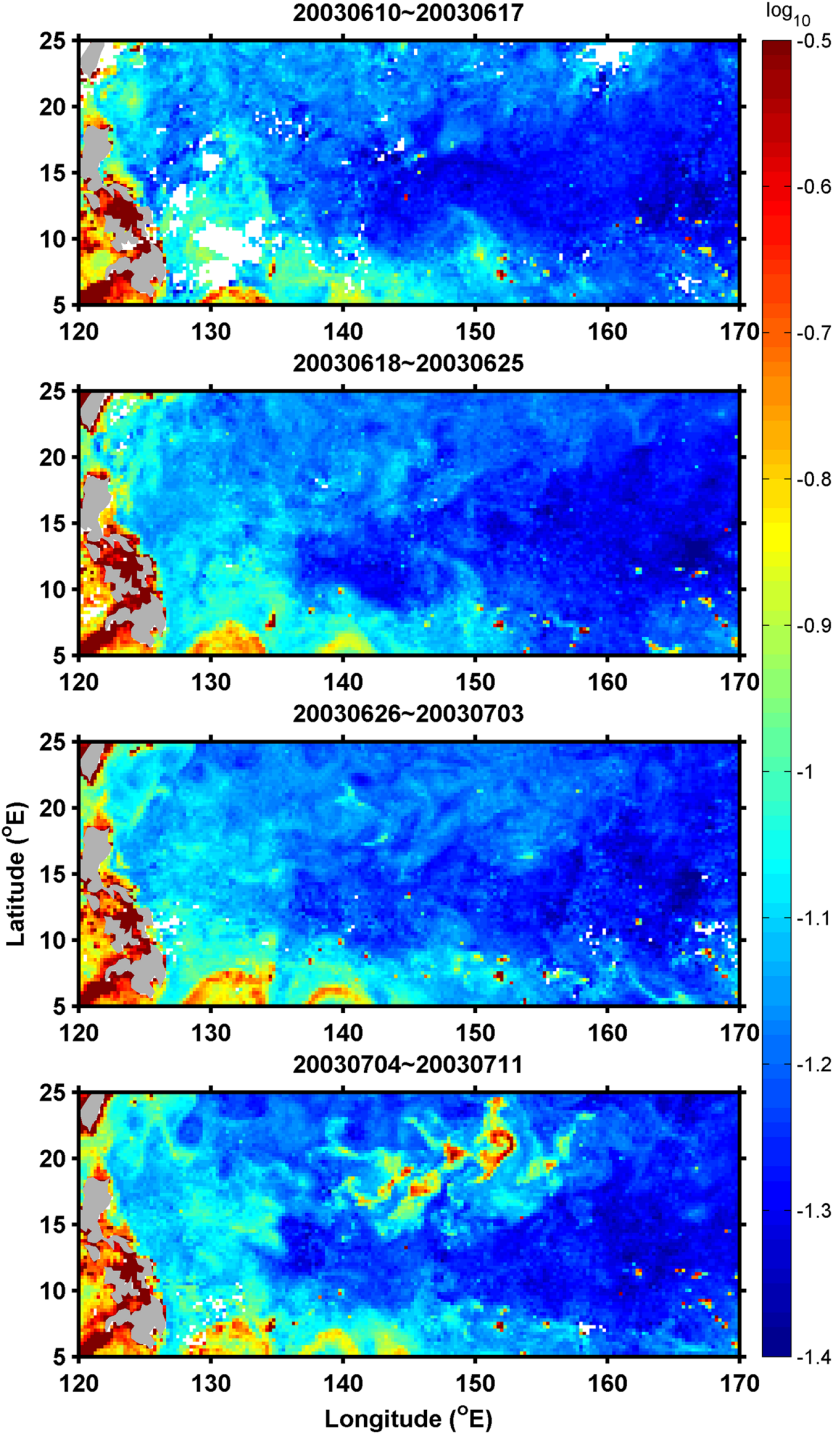
Ocean color images (log_10_ of Chl, mg/m^3^) from June 10 to July 11 in 2003.

The high Chl concentrations at the southern part of the eddies in Fig. 4 show a possible source of nutrients, particularly phosphate, coming from the NEC, as indicated in the northward advection of the Chl in the vicinity of 130°E in early June 2003 (Fig. 6). The northward advection of the Chl corresponds to the NEC bifurcation that shifted farther north in 2003 than in other years^22^. In the wNPSG, there is a large meridional gradient in the surface phosphate concentration in summer^23, 24^. The subtropical region between 17°N and 26.5°N suffers a severe phosphorus limitation with surface phosphate concentrations < 3 nM. Outside this region, the surface phosphate concentrations are at least three times higher (>10 nM) in the waters south of 17°N and north of 26.5°N^23, 24^. The near-complete consumption of phosphate in the subtropical region is linked to active nitrogen fixation by a number of diazotrophs, particularly nanoplanktonic cyanobacteria^24^.

**Figure 7:**
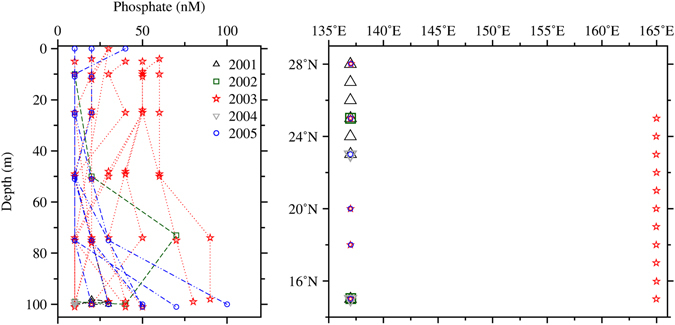
Vertical profiles of phosphate concentrations measured during the month of July by Japan Meteorological Agency from 2001 to 2005 within the region 15–28°N and 135–165°E (left), with corresponding locations (right).

The strong stirring by the eddy currents at the southern edge of the bloom likely encapsulated the high-phosphate waters from the northern front of the NEC to the bloom area. Indeed, the surface phosphate concentrations observed by the Japan Meteorological Agency (JMA) cruises within the bloom region were higher (>50 nM) in July 2003 than in other years from 2001 to 2005 for the same period (Fig. 7). Hence, the additional input of phosphate may have enhanced the nitrogen fixation activity in the subtropical region, further sustaining the bloom.

### Possible Deepening of Vertical Mixing

**Figure 8:**
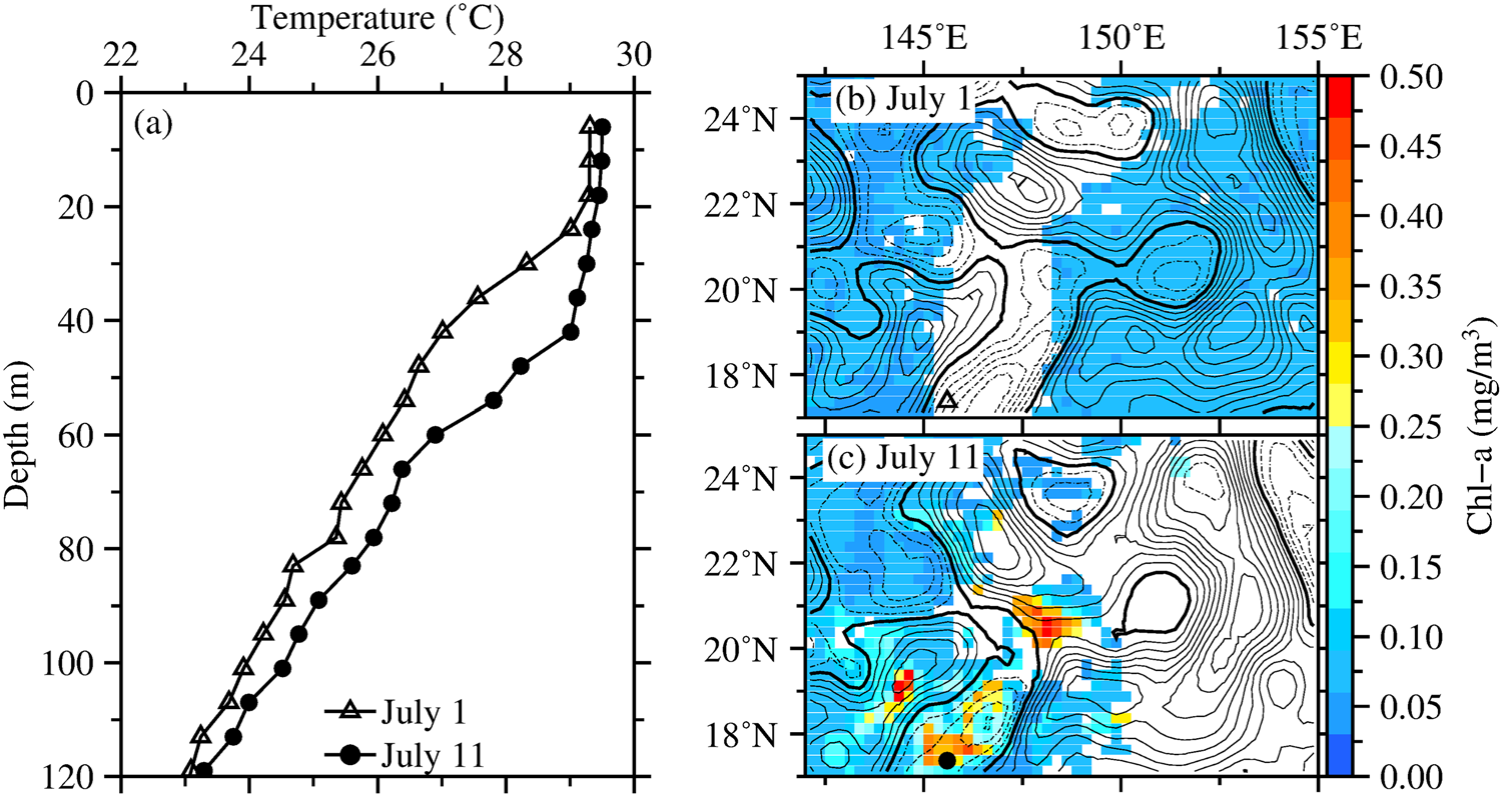
(**a)** Temperature profiles of Argo Q5900121 measured on July 1 (triangle) and July 11 (circle). (**b**) SSHAs (contour interval: 4 cm, dashed: negative, solid: positive, bold solid: zero) overlaid on Chl (colors, units in mg/m^3^) on (**b**) July 1 and (**c**) July 11, 2003. Triangle and circle symbols in (**b**) and (**c**) indicate the location of Argo Q5900121, respectively. This figure was generated using open source Generic Mapping Tools^33^ (Ver. 5.3.0 http://gmt.soest.hawaii.edu/).

**Figure 9:**
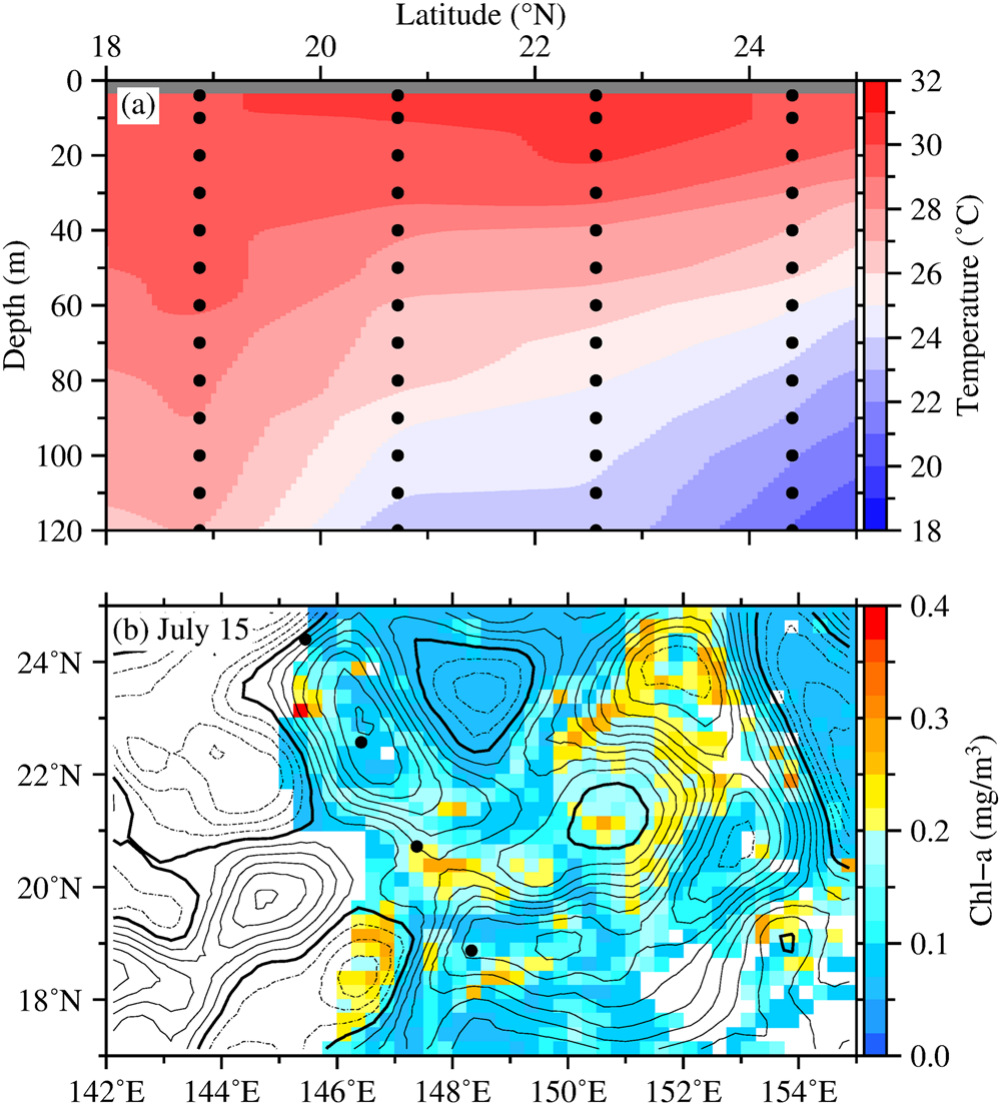
(**a**) Contour plot of temperature measured by CTD H9TO on July 15. Black circles indicate the actual depth of each observation. (**b**) SSHAs (contour interval: 4 cm, dashed: negative, solid: positive, bold solid: zero) overlaid on Chl (colors, units in mg/m^3^) on July 15. Black circles indicate the locations of CTD H9TO. This figure was generated using open source Generic Mapping Tools^33^ (Ver. 5.3.0 http://gmt.soest.hawaii.edu/).

Temperature profiles measured by Argo float (ID Q5900121) and shipboard CTD (ID H9TO) within the bloom area (Figs 8 and 9) show the variability of the vertical mixing during the bloom occurrence. Temperature profiles measured by the Argo float show an increased in the mixed layer depth from July 1 to July 11 (Fig. 8a), corresponding to the increase in the surface Chl concentration from 0.06 mg/m^3^ to 0.30 mg/m^3^ (Fig. 8b,c). The 29 °C isotherm surface obtained from the CTD profiles from south to north also show a deepening in the mixed layer down to 60 m at approximately 19°N (Fig. 9a), roughly corresponding to the high Chl concentration at the surface (Fig. 9b). This co-variability of the Chl concentration and vertical mixing depth implies a correlation between a deeper vertical mixing and higher surface Chl concentration in a nutrient-limited regime^25^. Thus, the deepening of the vertical mixing shows a possible uplifting of the nutrients supplied from the deeper depth to the sunlit layer^26^. This result agrees with the SSH-Chl correlation analysis of *Gaube et al*.^27^, suggesting that the high Chl concentration in the NPSG may also be maintained by the eddy impacts on the mixed-layer depth.

### Atmospheric Deposition

The monthly mean AOT, a proxy for atmospheric deposition, within the strongest bloom region (145–155°E and 17–25°N) shows that the atmospheric deposition during summer 2003 was rather weak compared to the summertime values in other years (Fig. 2a). In addition, no bloom was recorded in summer 2011 with the strongest dust deposition. The monthly mean AOT values are usually high in spring and summer and low in winter, which has an inverse relationship with the Chl concentrations, with the exception of 2003. In addition, the monthly mean AOT values were less than 0.2, showing that aerosol deposition was not strong in the bloom region. This condition illustrates that atmospheric deposition is unlikely to have been the mechanism triggering or driving the summer 2003 bloom.

## Discussion

This study reports the largest summer phytoplankton bloom ever recorded in the wNPSG. The bloom emerged as eddy features in the space domain and varied with eddies in the time domain. Our findings show that the eddies were responsible for the bloom mainly via horizontal stirring with anomalous phosphorous supplied from the NEC. The extremely strong eddy currents in summer 2003 swiftly spread the high Chl waters to the east and west with a speed faster than that of the eddy westward motion. Our findings differ from the current understanding of eddy-induced blooms that are westward-propagating and driven by the westward eddy motion. In addition, coinciding with the northward extension of the high-phosphate NEC waters, strong eddy stirring could have encapsulated the high-phosphate waters within the bloom area, further sustaining the blooms. The absence of the bloom when the aerosol measurements were high indicates that atmospheric deposition did not contribute to macronutrient loading to the bloom area. The extraordinary 2003 summertime bloom not only resulted in an increase in Chl six times that of the mean values but also collectively fixed an additional five teragrams of carbon during the one-month bloom period. However, the concomitant increase in the *p*CO_2_ during the bloom period indicates that this additional input of carbon was rapidly consumed and respired as CO_2_.

## Methods

All satellite observations used in this study cover a common period from 1998–2014. Daily, eight-day, and monthly Chl and AOT at 1/4° resolution were obtained from the European Space Agency’s GlobColour project (http://www.globcolour.info), which provides a merged Chl product with good coverage from sensors such as SeaWiFS, MERIS, MODIS-Aqua, and VIIRS. Daily altimetry SSH-anomalies (SSHAs) at 0.25° resolution were obtained from the Archiving, Validation, and Interpretation of Satellite Oceanographic (AVISO) data (http://www.aviso.altimetry.fr). For data consistency, the daily SSHAs were averaged at 8-day intervals according to the dates of the Chl. The geostrophic velocity anomalies (u′, v′) were derived from the zonal and meridional gradients of the 8-day SSHAs^2^, and the EKE was estimated as follows: 0.5*[(u′)^2^ + (v′)^2^]. The anomalies of the Chl in this study refer to deviations of the Chl concentration from the long-term mean of monthly means. The net primary production (NPP) rates were calculated based on the Vertically Generalized Production Model (VGPM)^28^. The VGPM includes Chl, photosynthetic active radiation from GlobColour, and sea surface temperature (SST) from Optimum Interpolation SST^29^ as input parameters. The *in situ* CTD, Argo, and drifter observations were obtained from the NOAA’s Atlantic Oceanographic and Meteorological Laboratory (http://www.aoml.noaa.gov/). The IDs of the concomitant CTD and Argo data are H9TO and Q5900121, respectively. The drifter data were used to calculate the eddy-current speed. The *in situ* nutrient data measured by the Japan Meteorological Agency were obtained via NOAA’s World Ocean Database 2013^30^ (https://www.nodc.noaa.gov/OC5/WOD/pr_wod.html).

Composite analysis was adopted to show mean distributions of the Chl concentration and NPP in cyclonic and anticyclonic eddies. The SSHA data within 135–165°N and 15–28°N were selected to track the eddy centres for the composite analysis. The eddy centre was defined by the maximum and minimum SSHAs for the anticyclonic and cyclonic eddies, respectively. For the cyclonic-eddy composite, a zonal distance between the maximum SSHAs at the west and east of each cyclonic eddy was obtained to normalize the x- and y-axes of the maps. We selected every cyclonic eddy with the SSHA difference above 0.08 m between the eddy-centre SSHA and the maximum SSHAs at its sides along the eddy-centre latitude. The 0.08 m value was selected to be twice as large as the SSH accuracy of approximately 0.04 m to avoid errors in satellite-altimetry measurements^31^. The approach was the same for the anticyclonic-eddy composite, but the SSHAs at the west and east of the eddies were minimum values. For averaging purposes, all maps of the SSHAs and Chl concentration corresponding to the selected eddies were centred on the eddy cores and normalized by the defined zonal distance. The *p*CO_2_ data were obtained from the National Institute for Environmental Studies^32^ (http://soop.jp/).

### Data Availability

The original datasets used in this study can be obtained from different data centers noted in the “Methods” section. The post-processed datasets generated during and analysed during this study are available from the corresponding author on reasonable request.
